# Effect of Local Anesthesia at the Bone Collection Site of Iliac Bone Grafting on Autonomic Nervous System Activity and Circulatory Dynamics in Alveolar Cleft Treatment

**DOI:** 10.1155/prm/1837287

**Published:** 2026-04-10

**Authors:** Kaoru Yamashita, Shusei Yoshimine, Akari Uto, Minako Uchino, Toshiro Kibe, Mitsutaka Sugimura

**Affiliations:** ^1^ Department of Dental Anesthesiology, Field of Oral and Maxillofacial Rehabilitation, Advanced Therapeutics Course, Graduate School of Medical and Dental Sciences, Kagoshima University, Kagoshima, Japan, kagoshima-u.ac.jp; ^2^ Department of Oral and Maxillofacial Surgery, Field of Oral and Maxillofacial Rehabilitation, Advanced Therapeutics Course, Graduate School of Medical and Dental Sciences, Kagoshima University, Kagoshima, Japan, kagoshima-u.ac.jp

**Keywords:** anesthesia, autonomic nervous system, clinical implications, iliac bone grafting, ropivacaine

## Abstract

**Background:**

In alveolar bone graft surgery, local administration of ropivacaine at the iliac bone donor site is expected to have an analgesic effect. However, its effects on autonomic nervous system activity and circulatory dynamics are unknown.

**Aims:**

This study aimed to assess the effects of local ropivacaine administration to the iliac region under general anesthesia by analyzing changes in autonomic nervous system activity and circulatory dynamics.

**Methods:**

We retrospectively reviewed medical records of patients who underwent iliac bone grafting under general anesthesia between May 2021 and December 2022. At the end of surgery, ropivacaine was administered locally in the iliac region. Heart rate variability (HRV), heart rate (HR), and systolic blood pressure (SBP) were analyzed 5 min prior to (baseline) and 5 min following (postadministration) ropivacaine administration.

**Results:**

Seventeen patients were included in the analysis. The low/high frequency ratio was significantly elevated after ropivacaine administration compared with baseline (*p* < 0.05). No significant changes were observed in high frequency, HR, or SBP (*p* < 0.05).

**Conclusion:**

Local administration of ropivacaine in the iliac region under general anesthesia resulted in detectable neurological changes, as evidenced by HRV analysis, though conventional circulatory parameters remained unchanged. HRV may provide sensitive detection of perioperative stress beyond what is captured by hemodynamic measures alone.

**Summary**
 a. What is already known about the topic?
 In bone grafting, postoperative pain at the bone graft site exceeds that at the receiver site. Topical administration of ropivacaine is expected to provide analgesia, but there is concern about circulatory fluctuations during administration. There are currently no reports analyzing how ropivacaine influences cardiac autonomic nervous activity and circulatory dynamics.
 b. What new information does this study add?
 In this study, subcutaneous administration of 0.75% ropivacaine increased sympathetic nervous activity, but the increase was not sufficient to significantly affect circulatory dynamics.

## 1. Introduction

Cleft lip and palate are common congenital abnormalities requiring multiple corrective surgeries [1]. Alveolar bone grafting serves to restore the continuity of the alveolar ridge, thereby enabling the proper eruption of teeth in individuals with this anomaly [1]. In bone grafting, postoperative pain at the bone graft site has been reported to be greater than that at the receiver site [2].

Postoperative pain management after iliac bone graft harvesting includes administering intravenous acetaminophen or flurbiprofen axetil, narcotic analgesics, and nerve blocks [1, 2]. The pain management method of choice is intravenous acetaminophen, given its safety profile in pediatric patients.

Nonetheless, in several cases, analgesia may prove inadequate [3]. Nonsteroidal anti‐inflammatory drugs can potentially cause gastric disturbances, renal dysfunction, and prolonged bleeding owing to platelet inhibition [4]. Narcotic analgesics are associated with postoperative respiratory depression [5], and nerve blocks are associated with complications such as vascular puncture, bleeding, and nerve damage [6]. Furthermore, continuous intravenous ropivacaine infusion through a catheter raises concerns about catheter‐related mechanical problems such as catheter occlusion and local anesthetic leakage around the catheter [7].

Local administration of ropivacaine may help manage pain at the extraction site; however, significant concerns remain regarding intraoperative complications like alterations in autonomic function and hemodynamics, characterized by increased heart rate (HR) and blood pressure associated with sympathetic excitation. Autonomic activity can be assessed by heart rate variability (HRV) analysis, and attempts have been made to monitor autonomic activity and circulatory dynamics during treatment [8–11].

Nevertheless, as far as we are aware, no previous research has investigated the effects of ropivacaine on cardiac autonomic nervous system activity and circulatory dynamics during general anesthesia. Furthermore, no clinical studies were identified in past reports that specifically quantified these effects when administered to the bone harvesting site during pediatric iliac crest bone graft surgery.

In this study, we aimed to investigate the effects of local iliac ropivacaine anesthesia, administered under general anesthesia, on the autonomic nervous system activity and circulatory dynamics.

## 2. Materials and Methods

### 2.1. Study Design/Sample

This retrospective study received approval from the Clinical Ethics Review Committee of Kagoshima University Hospital (no. 220324), with informed consent waived due to the study’s retrospective approach. Nevertheless, patients were informed about the potential use of their data in research. The sample size was estimated using a power analysis (*α* = 0.05 and *β* = 0.2) conducted with G∗Power software. Although 17 subjects were required according to the calculation, we set the target enrollment at 20 to accommodate potential attrition. Seventeen patients with alveolar clefts who underwent alveolar bone grafting under general anesthesia between May 2021 and December 2022 were ultimately included. Relevant information was extracted from anesthesia records, operative notes, and electronic medical records. Patients lacking complete data were excluded from the analysis.

### 2.2. Variables

The measurements of HRV, systolic blood pressure (SBP), and HR data obtained 5 minutes prior to the administration of local anesthesia served as baseline values for comparison.

### 2.3. Data Collection Methods

Measurements obtained 5 minutes following local anesthetic administration were evaluated as the “during” phase. Information regarding the administered dosage and intraoperative and postoperative adverse events, as well as postoperative pain management, was retrospectively collected from anesthesia charts and patient records.

### 2.4. Data Analyses

Anesthesia was induced either slowly or rapidly. Fluid therapy consisted of acetated Ringer’s solution with 1% glucose and a continuous infusion of remifentanil (0.3 μg/kg/min). Rocuronium (0.8 mg/kg) was administered to facilitate endotracheal intubation with a Macintosh laryngoscope. Mechanical ventilation was provided at a rate of 12 breaths per minute. To maintain anesthesia, sevoflurane (1.5%–2%) was combined with remifentanil at 0.1–0.5 μg/kg/min. Dexamethasone sodium phosphate and famotidine were used to prevent postoperative nausea and vomiting. As needed, sodium carbazochrome sulfonate and tranexamic acid were given for hemostatic support. Local anesthesia was achieved with 0.5% lidocaine containing 1:200,000 epinephrine, injected by the surgeon into the alveolar cleft and iliac bone harvest site prior to incision. Postoperative pain management was provided with intravenous acetaminophen (15 mg/kg) administered 30 min before surgery ended, flurbiprofen axetil (1 mg/kg) at surgery end, and local infiltration of 0.75% ropivacaine hydrochloride hydrate (3 mg/kg, max 75 mg) into the lower back after closure.

Assessment of the autonomic function involved analyzing HRV parameters from continuous intraoperative ECG data. HF (0.15–0.4 Hz) reflected parasympathetic activity, while LF (0.04–0.15 Hz) represented both sympathetic and parasympathetic influences. The LF/HF ratio, HF, and HR served as indicators for autonomic balance. Analysis was conducted using MemCalc‐Mackin2 (GMS, Tokyo, Japan). SBP was intermittently or noninvasively measured every 2 minutes as a circulatory indicator.

Statistical analysis was executed with GraphPad Prism, Version 9 (GraphPad Software, San Diego, CA), employing the Wilcoxon signed‐rank test. A *p* value < 0.05 was considered to denote statistical significance.

## 3. Results

Of the 20 eligible patients, three were excluded due to missing data. Thus, data from 17 patients were included in the final analysis. The mean age, height, weight, and total amount of local anesthesia (ropivacaine) used were 9.89 ± 0.19 years, 135.01 ± 1.92 cm, 29.79 ± 1.46 kg, and 9 ± 1.62 mL, respectively (Table 1).

**TABLE 1:** Patients’ physical characteristics and local anesthesia.

	

Age (years)	9.89 ± 0.19
Height (cm)	135.01 ± 1.92
Weight (kg)	29.79 ± 1.46
Local anesthesia (lidocaine) (mL)	6.9 ± 1.19
Local anesthesia (ropivacaine) (mL)	9 ± 1.62
Male/female sex	8/9

*Cleft type*	
Unilateral cleft lip and alveolus	2
Unilateral cleft lip and palate	12
Bilateral cleft lip and alveolus	0
Bilateral cleft lip and palate	3

In Figure 1, the LF/HF, HF, HR, and SBP were presented as ratios comparing postoperative to preoperative measurements. A significant increase in the LF/HF ratio was observed during drug administration compared with the period preceding administration (*p* < 0.05, Figure 1(a)). In contrast, no statistically significant changes were found in HF, HR, or SBP between the preadministration and during‐administration time points (Figures 1(b), 1(c), and 1(d)). Table 2 shows LF/HF, HR, and SBP values for individual patients before and during ropivacaine administration.

FIGURE 1Comparison of LF/HF, HF, HR, and SBP during and before the administration of local anesthesia (ropivacaine). LF/HF, HF, HR, and SBP are expressed as ratios relative to the preoperative values during the administration of ropivacaine. LF, low frequency; HF, high frequency; HR, heart rate; SBP, systolic blood pressure. (a) Comparison of LF/HF during and before the administration of local anesthesia (ropivacaine). ^∗^ *p* < 0.05. (b) Comparison of HF during and before the administration of local anesthesia (ropivacaine). ^∗^ *p* < 0.05. (c) Comparison of HR during and before the administration of local anesthesia (ropivacaine). ^∗^ *p* < 0.05. (d) Comparison of SBP during and before the administration of local anesthesia (ropivacaine). ^∗^ *p* < 0.05.

**Figure:**
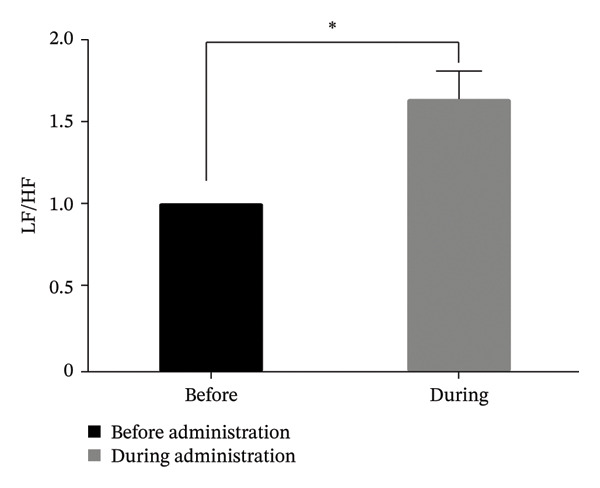
(a)

**Figure:**
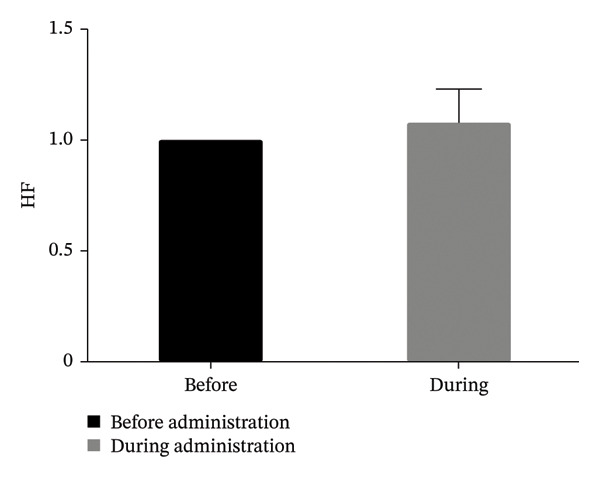
(b)

**Figure:**
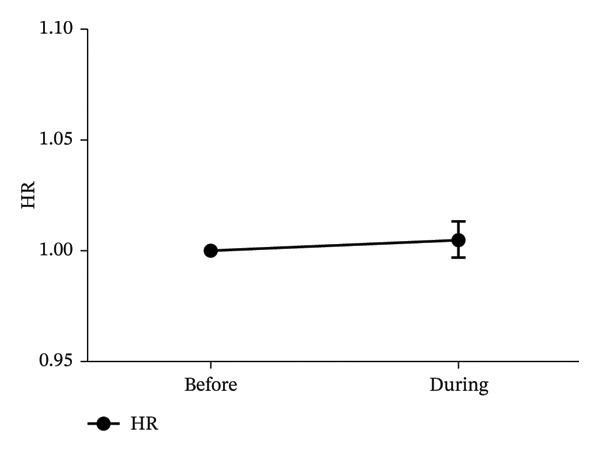
(c)

**Figure:**
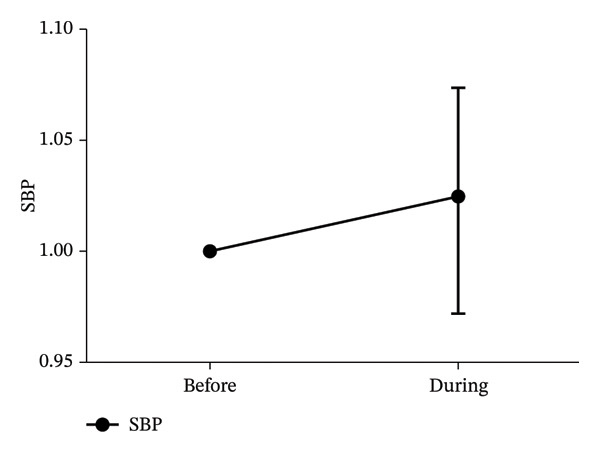
(d)

**TABLE 2:** LF/HF, HR, and SBP values for individual patients before and during ropivacaine administration.

LF/HF		LF (ms×ms)		HF (ms×ms)		HR (bpm)		SBP (mmHg)	
Before	During	Before	During	Before	During	Before	During	Before	During
4.71	6.99	78.19	85.21	16.60	12.19	77.4	78.3	104	108
1.94	2.13	227.45	274.40	117.24	128.83	73.2	72.7	84	91
9.86	18.9	108.16	95.48	10.97	5.05	79.9	81.2	83	82
0.15	0.22	5.17	5.54	34.46	25.17	75.8	76.4	88	94
0.58	0.65	59.36	93.37	102.35	143.65	65	65.5	78	82
1.44	2.83	112.92	235.50	78.42	83.21	74.1	73.2	88	90
0.51	1.55	115.77	150.35	227.00	97.00	82.6	89.2	96	97
1.32	2.15	10.65	12.17	8.07	5.66	81.9	80.5	93	91
7.15	26.24	54.19	757.98	7.58	28.89	86.0	83.6	82	96
1.04	1.36	51.03	58.90	49.07	43.31	63.6	64.9	96	98
1.37	2.27	5.24	6.79	3.82	2.99	84.6	85	91	86
0.27	0.3	46.90	43.37	173.69	144.57	75.8	76	84	84
1.85	2.1	1301.82	1949.67	703.69	928.41	94.1	94.6	87	88
1.33	2.14	119.65	141.88	89.96	66.30	77.3	75	86	84
0.77	0.87	22.13	16.19	28.74	18.60	84.1	81.5	80	79
0.44	0.47	13.00	21.76	29.55	46.30	82.9	81.4	85	84
1.15	1.64	24.40	28.56	21.22	17.42	68.3	70.7	94	91

## 4. Discussion

The 0.75% ropivacaine increased LF/HF but did not significantly alter circulatory dynamics. No intraoperative or postoperative complications were noted. This increased LF/HF may be a symptom of pain caused by needle insertion or the body’s stress response [9]. In our earlier study, we demonstrated that HRV analysis offers an objective means of assessing stress in pediatric patients, which can be challenging to evaluate through conventional interviews [12]. Furthermore, HRV analysis has been reported to potentially visualize stress that is difficult to assess solely through hemodynamic parameters [12]. Similar to previous reports, LF/HF may reflect the patient’s internal stress level but is not necessarily reflected in blood pressure or HR [9].

Sympathetic nerves innervate not only the sinus node but also the myocardium, increasing myocardial contractility and cardiac output, resulting in hemodynamic changes. Because BP is determined by the relationship between cardiac output and peripheral vascular resistance, cardiac sympathetic nerve activity does not necessarily correlate with BP [9]. HR is determined by the balance between sympathetic and parasympathetic nervous activity, but the parasympathetic nervous system in humans is dominant [13]. This is considered one reason why HR did not change significantly despite sympathetic stimulation. Multimodal analgesia is desirable in patients with iliac bone grafts because of concerns regarding residual pain in the iliac region and oral cavity, which are the sites of bone extraction [2]. In addition, because effective treatment of acute pain may prevent the development of chronic pain symptoms [14], continuing proper pain management from the intraoperative to the postoperative period is paramount [14]. Distinguishing pain between the extracted bone area and the surgical site in the oral cavity is considered difficult in children [15–19]. In our study as well, pain assessment proved difficult.

Previous studies involving adults have reported significant reductions in pain scores and morphine requirements with continuous postoperative local anesthetic infusion [20, 21]. Although analgesic efficacy of a continuous intravenous infusion of 0.2% ropivacaine in the iliac region has been reported in pediatric patients [22], concerns remain regarding complications such as infection from catheter placement and bleeding or neuropathy associated with the procedure. Nerve blocks can cause cardiac arrest through intravascular misinfusion [23]. However, there have been no reports on the effects of local ropivacaine administration on intraoperative autonomic nervous system activity and circulatory dynamics or on intraoperative and postoperative complications.

In this study, we analyzed HRV and hemodynamics during ropivacaine administration under general anesthesia. Following the guidelines, autonomic nervous system activity during local anesthesia was measured every 5 min [24]. In this study, we used data recorded 5 min before ropivacaine administration as the control condition and those recorded 5 min thereafter as the “during” condition. In this study, the anesthesia maintenance medication was the same, and the procedure was the same, with suturing occurring 5 min prior to administration. Furthermore, no new medications other than anesthetics were administered 5 min prior to the administration of local anesthesia.

Ropivacaine was initially injected at the maximum possible dose: within 10 mL of 0.75% ropivacaine, not exceeding the maximum dose (3 mg/kg). This was because the size of the skin incision and the amount that could be administered varies in the surgical field. Pain management with narcotics, such as morphine, may help achieve analgesia; however, narcotics can induce PONV and respiratory depression, restricting their use [5]. Local continuous intravenous infusion of ropivacaine may reduce narcotic use and the incidence of PONV [6]. This technique may be favorable compared with previously described techniques for pediatric patients undergoing iliac bone grafting. Sevoflurane has been reported to decrease HRV parameters [25, 26] and remifentanil to increase parasympathetic nervous system activity [27]. Although anesthetics and narcotics were maintained at constant concentrations at the time of data acquisition, the effects of anesthetics and narcotics on autonomic nervous system activity over time cannot be ruled out.

This study had some limitations. First, this was a retrospective study; therefore, it was not possible to match the amount of local anesthetic used. Second, comparisons with a group not administered any local anesthetic were not performed. Third, this study did not evaluate analgesic effects. Fourth, this study included a relatively small sample size, which may have resulted in insufficient statistical power. Therefore, the lack of statistically significant differences in outcome measures such as circulatory parameters does not necessarily indicate the absence of clinical or physiological effects.

## 5. Conclusions

In this study, we could monitor neurological changes during ropivacaine administration at the end of suturing in the bone collection area under general anesthesia. HRV analysis may have detected stress that is difficult to assess using circulatory dynamics alone.

## Author Contributions

Kaoru Yamashita: contributed to conception, design, data acquisition, analysis, and interpretation, performed all statistical analyses, drafted, and critically revised the manuscript. Shusei Yoshimine: contributed to conception, design, data acquisition, and interpretation, and drafted the manuscript. Akari Uto: contributed to conception, design, data acquisition and interpretation, analysis, and critically revised the manuscript. Minako Uchino: contributed to conception, design, data acquisition, and interpretation, analysis, and critically revised the manuscript. Toshiro Kibe: contributed to conception, design, data acquisition, and interpretation, and critically revised the manuscript. Mitsutaka Sugimura: contributed to conception, design, data acquisition, and interpretation, and critically revised the manuscript.

## Funding

This work was supported by the Japan Society for the Promotion of Science, Tokyo (KAKENHI grant no. JP21K17200).

## Disclosure

We presented a part of this paper at a conference of FADAS under the title “Effects of local anesthesia at the bone harvest site for iliac bone grafting on autonomic nervous activity and hemodynamics in the treatment of patients with alveolar clefts.” https://kokuhoken.net/jdsa/publication/file/journal/51-abstract/p209_FADAS.pdf. All authors gave their final approval and agree to be accountable for all aspects of the work.

## Conflicts of Interest

The authors declare no conflicts of interest.

## Data Availability

The data that support the findings of this study are available on request from the corresponding author. The data are not publicly available due to privacy or ethical restrictions.
